# Insight into structural properties of polyethylene glycol monolaurate in water and alcohols from molecular dynamics studies†

**DOI:** 10.1039/c9ra09688d

**Published:** 2020-06-08

**Authors:** Ianatul Khoiroh, Sze Ying Lee, Mohsen Pirdashti, Ming-Jer Lee

**Affiliations:** Department of Chemical & Environmental Engineering, Faculty of Science and Engineering, University of Nottingham Malaysia Jalan Broga 43500 Semenyih Selangor Darul Ehsan Malaysia Ianatul.Khoiroh@nottingham.edu.my +60-3-8924-8017 +60-3-8924-8361; Department of Chemical Engineering, Faculty of Engineering and Science, Universiti Tunku Abdul Rahman Sungai Long Campus Kajang 43000 Selangor Malaysia; Chemical Engineering Department, Faculty of Engineering, Shomal University PO Box 731 Amol Iran; Department of Chemical Engineering, National Taiwan University of Science and Technology 43 Keelung Road, Section 4 Taipei 106-07 Taiwan

## Abstract

By means of molecular dynamics (MD) simulations, we explored the structural properties of polyethylene glycol monolaurate (PEGML) in water and in various aliphatic alcohols (methanol, ethanol, 2-propanol, 2-butanol, *tert*-butanol, and 1-pentanol). The PEGML and the alcohols were simulated using the optimized potentials for liquid simulations, all-atom (OPLS-AA) force field and water using the extended simple point charge (SPC/E) model. From the isothermal-isobaric (*NPT*, constant number of particles, constant pressure, and constant temperature) ensemble, we extracted the densities from the simulations and compared them with those from experimental results in order to confirm the validity of the selected force fields. The densities from MD simulations are in good agreement with the experimental values. To gain more insight into the nature of interactions between the PEGML and the solvent molecules, we analyzed the hydrogen-bonds, the electrostatic (Coulomb) interactions, and the van der Waals (Lennard-Jones) interaction energies extracted from MD simulations. The results were further strengthened by computing the solvation free energy by employing the free energy perturbation (FEP) approach. In this method, the free energy difference was computed by using the Bennet Acceptance Ratio (BAR) method. Moreover, the radial distribution functions were analyzed in order to gain more understanding of the solution behavior at the molecular level.

## Abstract 2

By means of molecular dynamics (MD) simulations, we explored the structural properties of polyethylene glycol monolaurate (PEGML) in water and in various aliphatic alcohols (methanol, ethanol, 2-propanol, 2-butanol, *tert*-butanol, and 1-pentanol).

## Graphical abstract

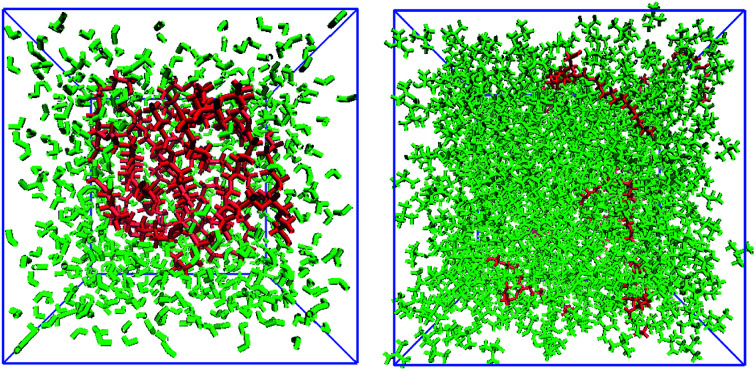

## 1 Introduction

Polyethylene glycols (PEGs) and their derivatives have widespread applications as specialty nonionic surfactants. These compounds are amphilic macromolecules with the general formula H–(CH_2_–CH_2_–O)_*n*_–OH where *n* is the average number of repeating oxyethylene groups. Primarily, they are prepared by addition of ethylene oxide onto aliphatic alcohols in the presence of suitable catalysts. Various surfactant homologs can be synthesized either by varying the number of ethylene oxide repeating units or the length of the alkyl chain of the alcohols. The versatility of these PEG-derived surfactants arises from their efficient solubility with water and most organic solvents such as methylene chloride, ethanol, toluene, acetone, and chloroform.^1,2^ Besides, PEGs are non-toxic, low in volatility, and biodegradable which make them a suitable choice as “green” solvents.^3–5^ These extraordinary properties are mainly due to the importance of the hydroxyl terminal groups in their polymeric chains. PEG functional structures are attractive in industrial uses since the repeating unit of PEGs is like an open crown ring ether, which gives peculiar interactions with some molecules or ions.^6^ Moreover, an active hydrogen attached to a heteroatom can be easily fine-tuned to form a reactive anion to other molecules or a surface. They belong to an important class of nonionic surfactants due to their low price, low toxicity and volatility, and biodegradability.^7–11^ PEG-based surfactants have been produced from the nanoscale to the macroscale industry and are widely used in household and cleaning products, agriculture, biotechnology, food industries, and pharmaceutical processes.^12–18^

When PEG-based surfactants are used in practice, they are generally mixed with other solvents to improve their performance. Among those additives, alcohols are the most frequently used to form microemulsions or various solubilized systems. For example, short to medium chain length alcohols have been used in the tertiary oil recovery process to enhance the stability and decrease the viscosity of the micellar system.^19–21^ As a result, the higher efficiency can be achieved and this contributes to the cost reduction of the overall process.

Similarly, surfactant/alcohol mixture can be used for *in situ* flushing agent for the remediation of aquifers contaminated with nonaqueous phase liquid.^22–24^ The ternary systems of alcohol–surfactant–water have been of particular interest for experimental and theoretical scientific studies. Likewise, the roles of alcohols on both equilibrium and dynamic properties of aqueous micellar solutions have been extensively reported in the literature, including critical micelle concentration, surface tension, counterion binding, aggregation number, and so forth.^25–27^ However, the specialized studies on the binary systems containing alcohol and surfactant are restricted to just a few examples. This study is important to obtain some solid basis of binary solution behavior in order to study rather complicated ternary systems.

Molecular dynamics (MD) simulation has established as a powerful tool to probe the microscopic structures and properties of surfactant systems. It is useful for the understanding of the physical basis of the structure–function relationship of macromolecules at the atomistic level. MD simulation reduces the experimental efforts and can provide crucial detail regarding the motions of particles as a function of time. Thus, it can be used to model the specific systems in such easier way than experiments on the actual system. In the present work, we attempted to investigate the solution behavior of polyethylene glycol monolaurate (PEGML) in water and in alcohols (methanol, ethanol, 2-propanol, 2-butanol, *tert*-butanol, and 1-pentanol), through MD simulation.

In terms of its commercial importance, the selected oligomer mainly served as emulsifier blends, thickener, resin plasticizer, emollient, opacifier, spreading agent, wetting and dispersing agent, and viscosity control agents.^28^ It also has applications in metalworking, pulp, paper, textile and as defoamers for latex paints. A systematic study and understanding of the physicochemical properties of PEG/alcohol mixtures is therefore of practical importance to further explore the great number of PEGs applications in various fields. A comprehensive knowledge of the relation between the structure and the properties of these mixtures, however, is lacking at this point.

The chemical characteristics of PEGML is analogous to that exhibited by lower chain monomers such as PEG as well as polyethylenoxide (PEO), dimethoxyethane (DME), polyvinylalcohol (PVA) in addition to surfactant such as sodium-dodecyl-sulfate (SDS), all of which have been extensively researched and hence forms the basis of understanding of the system. Behavior of PEGML in water can be correlated to that of aqueous 1,2-DME/DMP, where molecular dynamic simulations showed that the degree of DME/water hydrogen bonding is nearly independent of the DME conformer for dilute solutions and over a wide range of concentrations.^29–31^ However, in the studied simulations, the concentration is fixed hence conformation variation is not expected in the system. It was also deduced that DME intermolecular interactions are dominated by Lennard-Jones interaction while electrostatic interaction dominates in pure solvent, with solvent–solvent intermolecular energy decreasing with an increasing DME concentration as well as resulting in reduced DME–solvent hydrogen bonding (largely due to solvent–solvent interaction). Nonetheless, PEGML has hydrophilic hydroxyl group attached at one end in addition to 6 ether atoms hence hydrogen bonding with solvent is expected to be significant. Our main goal is therefore to gain a better understanding on the behaviors of PEGML in both aqueous and alcohol solvents, and to reveal details of their structure–function relationship and dynamic properties extracted from the atomistic simulations.

MD simulations were performed by using the freely available GROMACS software package (version 4.5.5).^32–34^ The density measurements, were also conducted and the results were compared with those the calculated values from MD simulations. To gain more insight to the nature of interactions between PEGML and the solvent molecules, the hydrogen-bonds, the electrostatic (Coulomb) interactions, and the van der Waals (Lennard-Jones) interactions energies were extracted and analyzed from MD simulations. The results were further strengthened by computing the solvation free energy for each binary system. Moreover, the radial distribution functions was analyzed in order to gain more understanding of the solution behavior at molecular level.

## 2 Methodology and simulation details

A single molecule of PEGML was built in ChemOffice program.^35^ A script written in Perl interpreter, MKTOP,^36^ was used to construct the initial topology of PEGML for GROMACS. The topology file generated was carefully checked and refinements were subsequently made. The OPLS-AA force field^37–39^ was selected to describe PEGML and the alcohols molecules, and the SPC/E (extended simple point charge) model was used for water molecules. Partial charges of PEGML were computed by the Austin Model 1 using bond charge correction (AM1-BCC)^40,41^ implemented in the HyperChem^42^ (version 8.0.7) program. The included file for topology (itp file) and calculated atomic charges are listed in Table S1 (ESI†) while the atomic numbering is given in Fig. S1.† Force field parameters for PEGML were obtained from the original OPLS-AA parameterization. The OPLS-AA topology and structure files for the alcohols, except for 2-propanol and 2-butanol, were taken from the Virtual Chemistry database.^43^ In the present work, the liquid simulation boxes of 2-propanol and 2-butanol were prepared according to Caleman *et al.*^44^ Density Functional Theory (DFT) calculations were performed in Gaussian 09 package^45^ to calculate the atomic charges for the last two alcohols. The Becke three-parameter exchange functional with the gradient-corrected correlation functional of the Lee–Yang–Parr (B3LYP)^46^ and 6-311G basis set were used in the DFT calculations.

One molecule of PEGML was first energy minimized and simulated in vacuum for 10 ns to obtain the optimized configurations as a starting structure. In binary systems, PEGML was simulated in the aqueous and alcohols solutions at 298.15 K. A total of 990 solvent molecules was used to solvate 10 molecules of PEGML, corresponding to the concentration of 0.01 of PEGML in mole fraction (*x*_1_). A cubic box type with periodic boundary conditions was applied in three directions of the Cartesian coordinates. To prepare simulation box, we first generated a 2 × 2 × 2 nm^3^ box containing a single molecule of PEGML optimized from the vacuum simulation. Then we stacked 2 × 2 × 2 of the molecule box and inserted additional 2 oligomer molecules to obtain a preliminary simulation box which contained 10 molecules of PEGML. This box was then scaled to the estimated volume of the custom 990 molecules of solvents added. As for pure PEGML simulation, we stacked 30 × 30 × 30 of the box containing one molecule of PEGML to obtain a final box containing 1000 molecules of PEGML.

The PEGML and solvated configurations were energy-minimized for 5 × 10^5^ steps using a steepest-descent method in order to remove the unfavorable contacts. A preliminary series of simulations was performed to ensure the equilibration of all the properties of the system. First, the unit cell was simulated under high pressure (100 bar) at 298.15 K for 100 ps in order to achieve a realistic liquid density. The systems then relaxed at atmospheric pressure (1 bar) for 100 ps. The systems were further stirred in an *NVT* (constant number of particles, constant volume, and constant temperature) ensemble at 800 K for 100 ps and followed by another 100 ps at 298.15 K. Finally, production simulations were carried out in the isothermal-isobaric (*NPT*, constant number of particles, constant pressure, and constant temperature) ensemble at 298.15 K and 1 bar for 55 ns. For each of the system, the results of the first 50 ns were dropped and the last 5 ns was used for analysis. The final size of the equilibrated box for the studied systems is given in Table 1. The Newton equation was solved using the leap-frog^47^ integrator with time step of 2 fs. A twin-range 0.9 nm cut-off was used for the short range electrostatic (Coulomb) interactions and truncated at 1.4 nm for the Lennard-Jones (van der Waals) interaction. The neighbors searching was updated every ten simulation steps at this distance. Long range electrostatic interactions were evaluated by smooth particle mesh Ewald (PME) method^48^ with cubic interpolation and maximum grid spacing of 0.125 nm. The reference temperature was controlled by chained Nose–Hoover thermostat^49,50^ with time constant for coupling of 1.0 ps. The Parrinello–Rahman pressure coupling barostat^51^ was chosen with compressibility set to 4.5 × 10^−5^ bar^−1^ with a relaxation time of 2.0 ps. The bonds in the molecules were constrained using the Linear Constraint Solver (LINCS)^52^ algorithm with a fourth order in the expansion of the constraint coupling matrix. The simulated trajectories were saved and written every 1 ps in the disk. In all cases, the potential energy was stable as a criterion for equilibration. The block averaging method^53^ was implemented to calculate the errors in the calculated properties.

**Table 1:** The description of the materials

Compound	*M*_w_/g mol^−1^	Source	Mass fraction purity
PEGML	400 (*n ca.* 5)	Sigma-Aldrich, USA	>0.99
Methanol	32.04	Sigma-Aldrich, USA	>0.998
Ethanol	46.07	Sigma-Aldrich, USA	>0.998
2-Propanol	60.10	Sigma-Aldrich, USA	>0.998
2-Butanol	74.12	Sigma-Aldrich, USA	>0.998
*tert*-Butanol	74.12	Acros, USA	>0.995
1-Pentanol	88.15	Sigma-Aldrich, USA	>0.998

As for the solvation free energy calculations, each simulation consisted of one solute PEGML solvated in 299 molecules of solvents at 298 K and 1 bar in a cubic box. The Lennard-Jones interactions are turned off between PEGML and solvents with the soft-core potential alpha and sigma being set to 0.5 and 0.3, respectively. The simulation was performed on 21 *λ* points between zero and one with an equidistant lambda spacing of 0.05. Finally, the free energy difference from each *λ* window was estimated using the Bennett Acceptance Ratio (BAR) method^54^ implemented in GROMACS. During the simulation, the temperature was handled *via* Langevin stochastic dynamics^55^ while the constant pressure runs the Parrinello–Rahman barostat^45^ with a time constant of 0.5 ps. Isothermal compressibility was set to 4.5 × 10^−5^ bar^−1^ to enforce pressure coupling. Each simulation was performed independently from the same initial configuration. For each *λ* value, the starting structure was first minimized using the steepest descent method for 5000 steps and minimized further by the Limited-memory Broyden–Fletcher–Goldfarb–Shanno (L-BFGS) algorithm^56^ for 5000 steps. A constant volume equilibration was performed for 100 ps followed by a constant pressure equilibration for 100 ps. Finally, a 500 ps production stage was run under *NPT* ensemble and used for analysis. All MD simulations were assigned a 0.9 nm cutoff radius for electrostatic interactions and 1.4 nm for Lennard-Jones interactions. The nonbonded interactions were updated every step. Analytic long-range corrections to energy and virial were applied and evaluated using the PME method.^48^ An alternative way to setup these binary simulation systems would be to use PackMol.

## 3 Results and discussion

Thermodynamic properties can be calculated from statistical thermodynamics based on the fluctuations in the *NPT* ensemble. These thermodynamic properties are obtained from a single MD simulation run at the desired state point, thus, give an elegant method to compute the thermodynamic properties. The validity of the selected force field in MD simulations can be checked by comparing the liquid densities from computational results with experimental values.^57–59^ The density predictions as a function of temperature at isobaric conditions may be obtained simply *via* MD simulations in *NPT* ensemble. It follows trivially from the mass of the system *M* divided by the system volume *V*:1
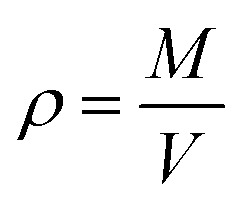


Densities were measured with an Anton Paar DMA-4500 vibrating-tube densimeter, Austria, with an uncertainty of ±5 × 10^−5^ g cm^−3^. The description of the materials used in this study is given in Table 1. Densities of pure PEGML and its mixtures as well as their comparison with the experimental data and their respective deviations are shown in Table 2. The given uncertainties were calculated by dividing the simulations into 5 blocks of time, where the average property was computed in each block followed by computing the corresponding standard error in the block average. The MD simulation result of the pure liquid PEGML at 298.15 K gives an underestimation of 0.8% from the experimental value. Moreover, the comparison between computational and experimental results for PEGML in alcohols (*x*_1_ = 0.01) exhibits an underestimation of 1.9% for PEGML + methanol, 1.0% for PEGML + 2-butanol, and 0.6% for PEGML + 1-pentanol; and an overestimation of 0.3% for PEGML + ethanol, 0.9% for PEGML + 2-propanol, and 4.6% for PEGML + *tert*-butanol, respectively. It is notable that water is immiscible in PEGML, thus, the density measurement could not be carried out for this system. The feasibility of the aqueous system was assessed by determining the total potential energy which was determined to be acceptable. Overall, the simulated density results of pure PEGML and its mixtures are in satisfactory agreement with the experimental values, thus, confirming the reliability of the selected force field in these MD simulations. To further confirm the suitability of AM1-BCC protocol, the partial charges were derived using restrained electrostatic potential (RESP) scheme provided by Multiwfn^60^ for oligomeric PEGML. The calculated charges are presented in Table S2 (ESI†) and they were subsequently tested to compute the density of PEGML with methanol. It is found that the result is satisfactory, *i.e.* the average absolute deviation (AAD) was found to be 1.3% for the density value extracted by using AM1-BCC and RESP charges, respectively.

**Table 2:** Simulated and experimental densities for pure PEGML and its binary systems at 298.15 K

System^a^	*ρ*_sim_/gcm^−3^	*ρ*_expt_ b/g cm^−3^	10^2^Δ*ρ*/*ρ*^c^	Box size/nm^3^
PEGML	0.9711 ± 0.0071	0.9789	0.8	3.57
M1	—	—	—	3.32
M2	0.7907 ± 0.0002	0.8062	1.9	4.23
M3	0.8016 ± 0.0002	0.7994	0.3	4.67
M4	0.8004 ± 0.0004	0.7930	0.9	5.10
M5	0.8019 ± 0.0001	0.8102	1.0	5.44
M6	0.8264 ± 0.0002	0.7901	4.6	5.38
M7	0.8126 ± 0.0001	0.8176	0.6	5.71

a M1: PEGML + water; M2: PEGML + methanol; M3: PEGML + ethanol; M4: PEGML + 2-propanol; M5: PEGML + 2-butanol; M6: PEGML + *tert*-butanol; M7: PEGML + 1-pentanol. The mole fraction of PEGML is 0.01 in each binary mixture.

b *u*(*ρ*) = 0.00005 g cm^−3^.

c Δ*ρ*/*ρ* = |*ρ*_sim_ − *ρ*_expt_|/*ρ*_expt_, where subscripts sim and expt are the simulated and the experimental values, respectively.

**Fig. 1:**
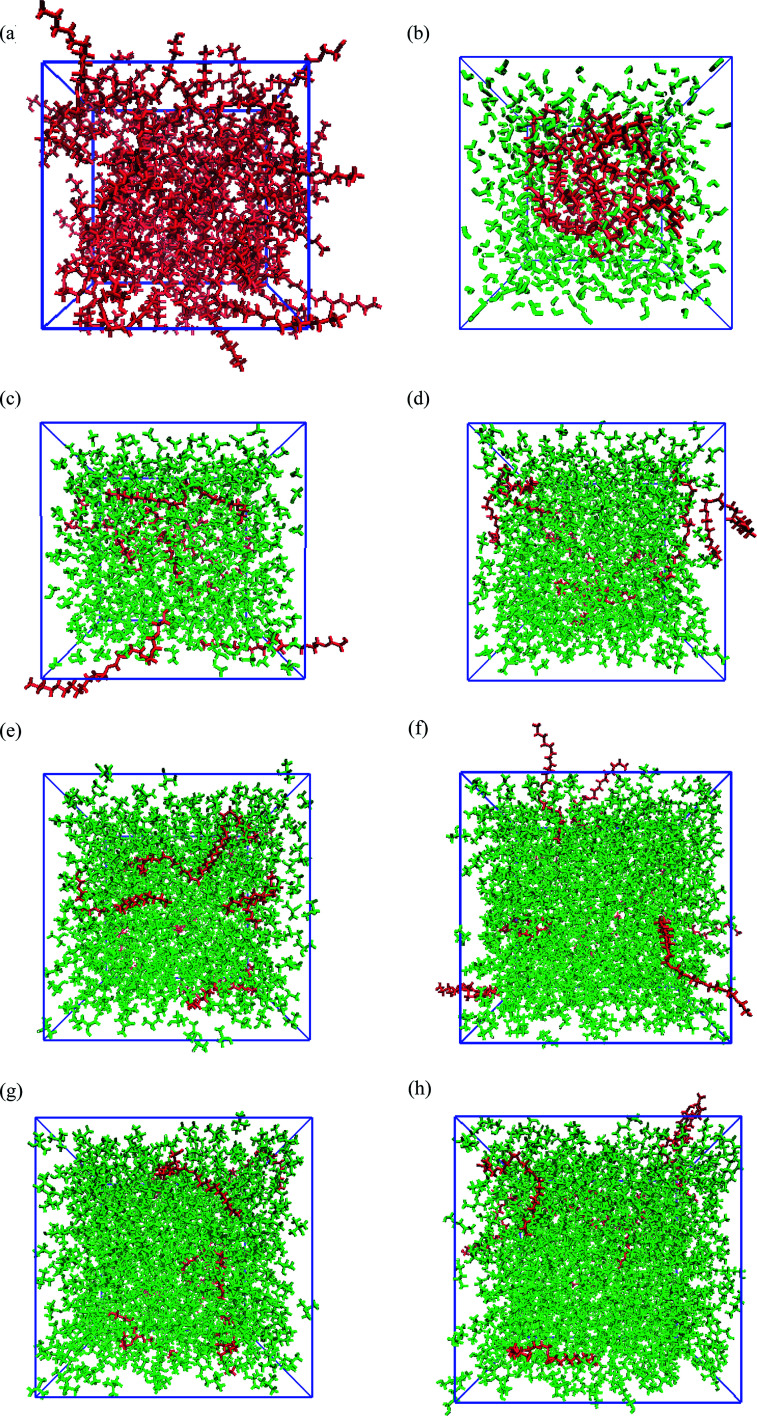
Snapshots for the configurations of PEGML (red) in the solvent (green) after 55 ns simulation: (a) PEGML; (b) PEGML + water; (c) PEGML + methanol; (d) PEGML + ethanol; (e) PEGML + 2-propanol; (f) PEGML + 2-butanol; (g) PEGML + *tert*-butanol; (h) PEGML + 1-pentanol. All solvent molecules were omitted for the ease of visualization.

Fig. 1 shows the snapshots of PEGML and its binary mixtures after 55 ns *NPT* simulations. These snapshots were rendered by using the Visual Molecular Dynamics (VMD)^61^ software. The hydrogen atoms, including the polar hydrogens, were depicted by white colour, carbon atoms by blue, and oxygen atoms by red. The shape and size of simulated PEGML in aqueous solution is given Fig. 1(a) in which the shielding of hydrophobic tails from water with the formation of aggregates was observed and can be clearly viewed from the given trajectory. The structure of an aggregate is compact and exposing the terminal polar (OH) and ethylene glycol (–CH_2_–CH_2_–O–) groups at the surface while the alkyl (carbon) chains, as expected, occupy the central core. In the aqueous solution, PEGML which can be classified as a non-ionic surfactant, is expected to orient its hydrophilic part toward the outside of the micelles. The driving force for the micellar formation is associated to the hydrophobic effect, which excludes non polar moieties from the water to the micelle structure. The effect of micellar organization predicted in the polymer–water ensemble was studied by Shang^62^ and he showed that polymer hydrophilic group resides at the hydrocarbon–water interface hence leading to a selective reduction in the hydrophobic contribution to the solvent-accessible surface area of the micelle, with the driving force mainly being hydrophobic interactions between the polymer alkyl groups. By observing the trajectory, it was also determined that the randomly positioned molecules of PEGML first aggregate in small clusters, which then come together to form a single micelle. It then undergoes restructuring, to finally achieve its final equilibrium arrangement.

The snapshots of PEGML with alcohols in which the normal distribution of alcohol solvents can be clearly observed from Fig. 1(c)–(h). The presence of alcohols alter micelle behavior, which decreases the minor radius of micelle and progressively breaks down hence prevents micellar formation, due to the ability of alcohols to solubilize the micellar core structure. Moreover, increased hydrophobicity of PEGML due to larger alkyl chain is also predicted to prevent micelle development in alcohols. On the other hand, investigations into the structural and thermodynamic properties of polyethylene terephthalate (PET) by Watanabe^63^ showed that intramolecular and intermolecular non-bonded contributions to the potential energy decreased in magnitude with increasing degree of polymerization, due to the diminished role of hydrogen bonding in the system, *i.e.* increased fraction of alcohol groups engaged in hydrogen bonding.

### Hydrogen bonds and intermolecular interactions

The calculated average number of hydrogen bonds 〈*N*_H-bonds_〉 for PEGML–solvent, PEGML–PEGML, and solvent–solvent, are reported in Table 3, while the electrostatic (Coulomb) and the Lennard-Jones (van der Waals) interaction energies are summarized in Table 4. The average number of hydrogen bonds per molecule was determined from the trajectories based on a geometrical criterion with a cutoff donor–acceptor (DA) distance of at most 0.35 nm and a cutoff donor–hydrogen–acceptor (DHA) angle of at most 30°. OH groups are regarded as donors and O as an acceptor. From Table 3, it is apparent that the average number of hydrogen bonds per PEGML with alcohol molecules is around 1 per molecule for all the series. Briefly, 〈*N*_H-bonds_〉 with methanol is 1.531. 〈*N*_H-bonds_〉 per PEGML with ethanol and 2-propanol are found to be similar, which are 1.175 and 1.116, respectively. Similarly, the average number of hydrogen bonds per oligomer molecule with 2-butanol, *tert*-butanol, and 1-pentanol are 0.700, 0.930, 0.938, respectively.

**Table 3:** The number of intramolecular hydrogen bonds and their corresponding lifetime between adjacent oligomer in solvents

System^a^	Interaction	Number of hydrogen bonds	Hydrogen bond lifetime (ps)
M1	PEGML–solvent	3.993	3.638
	PEGML–PEGML	0.059	4.477
	Solvent–solvent	1.760	4.332
M2	PEGML–solvent	1.531	2.027
	PEGML–PEGML	0.017	2.241
	Solvent–solvent	0.895	6.396
M3	PEGML–solvent	1.175	3.296
	PEGML–PEGML	0.017	3.092
	Solvent–solvent	0.900	10.812
M4	PEGML–solvent	1.116	1.431
	PEGML–PEGML	0.017	2.443
	Solvent–solvent	0.609	3.269
M5	PEGML–solvent	0.700	1.096
	PEGML–PEGML	0.010	2.242
	Solvent–solvent	0.246	1.474
M6	PEGML–solvent	0.930	3.968
	PEGML–PEGML	0.009	3.056
	Solvent–solvent	0.842	104.869
M7	PEGML–solvent	0.938	8.229
	PEGML–PEGML	0.016	2.923
	Solvent–solvent	0.877	36.045

a M1: PEGML + water; M2: PEGML + methanol; M3: PEGML + ethanol; M4: PEGML + 2-propanol; M5: PEGML + 2-butanol; M6: PEGML + *tert*-butanol; M7: PEGML + 1-pentanol.

**Table 4:** The electrostatic (Coulomb) and the van-der Waals (Lennard-Jones) energies obtained from the MD simulations for the interaction of (PEGML–PEGML) and (PEGML–alcohol)

System^a^	Coulomb/kJ mol^−1^		Lennard-Jones/kJ mol^−1^	
	PEGML–PEGML	PEGML–alcohol	PEGML–PEGML	PEGML–alcohol
M2	157.0	−338.5	−339.6	−1914.8
M3	148.7	−205.6	−380.2	−1951.0
M4	155.8	−60.9	−281.2	−2142.4
M5	156.9	−15.2	−268.5	−2212.2
M6	147.6	−105.5	−309.5	−2141.4
M7	158.7	−159.8	−276.8	−2275.8

a M2: PEGML + methanol; M3: PEGML + ethanol; M4: PEGML + 2-propanol; M5: PEGML + 2-butanol; M6: PEGML + *tert*-butanol; M7: PEGML + 1-pentanol.

PEGML–solvent hydrogen bond (Table 3) interaction appears to occur much more frequently as compared to the solute–solute (PEGML–PEGML) case, showing a decreasing trend with increasing carbon chain length in the alcohol molecule. This trend was also observed by van der Spoel^64^ and can be attributed to the steric hindrance factor, whereby the larger space requirements of larger size alcohols *i.e.* ethanol and propanol compared to water that prevents further molecules from reaching the bonding sites.^65^ In addition, Hezaveh *et al.*^66^ corroborated this and also noted that the presence of increasing methyl group in the backbone chain prevented the formation of strong hydrogen bonds with the solvent. However it is noteworthy that water shows the highest degree of hydrogen bonding although it is immiscible in water. This can perhaps be explained by the findings of Desmukh *et al.*^67^ which was corroborated by Vrhovsek *et al.*,^68^ from which it can be theorized that the hydrophobic CH_2_–CH_2_ group in the backbone of PEGML are encaged by water molecules, thereby preventing it from interrupting the hydrogen bond network formed between the ether oxygen atoms on the backbone chain and the hydrogen donor of the solvent. It can be further construed that for all solvent systems, it's the ether oxygen–solvent hydrogen that predominates hydrogen bonding as compared to the terminal group bonding and this is verified by observing the PEGML–water system, whereby three out of the four hydrogen bonds formed per PEGML molecule is with the ether oxygen atom. The importance of the ether group was substantiated based on the findings of Heymann *et al.*,^69^ whom observed the formation of water bridges *i.e.* simultaneous hydrogen bonding to two ether atoms, as well as that of Fenn *et al.*,^70^ whom found that for a 10 : 1 concentration (similar to the present study), at least 2 hydrogen bonds between the solvent and the solute ether oxygen atoms. However they also discerned that not all ether oxygen atoms accepts hydrogen bonding with water hydroxyl group, which partially explains the non-bonding of the remaining three ether as well as the epoxy oxygen atoms, possibly due to the steric hindrance factors seen earlier for organic alcohols as well as the alignment of the backbone chain during micelle formation for PEGML–water system.

On the other hand, solvent–solvent hydrogen bonding demonstrates lower degree of hydrogen bonding as compared to solute–solvent bonding. PEGML–water system shows highest inter-solvent bonding with 1.760 bonds formed per solvent. Again the decreasing trend observed can be explained *via* the steric hindrance factor, with hydrophobic methyl groups preventing proximity below the required distance (0.35 nm) required for bond formation. However it must be noted that this high degree of hydrogen bonding is only valid when the solvent concentration exceeds 0.8 M, as proven by Raabe and Kohler.^71^

The hydrogen bond lifetimes for different molecule (Table 3) pairs show wildly differing trends. Previously it was found that PEGML–PEGML hydrogen bonding is relatively insignificant however a decreasing trend for bond stability was observed, from 4.477 ps for water to 2.923 ps for 1-pentanol. This shows that although PEGML–PEGML hydrogen bonding is five times less likely to occur in the 1-pentanol mixture, the resultant bonding is able to resist rotational and torsional motion, which causes bond breakage, for almost two times as long. Van der Spoel explained this phenomena *via* enthalpy, whereby there exists an enthalpic penalty for transitioning from bound to unbound state in increasing non-polar environments.^64^ The solute–solvent hydrogen bond lifetime however showed an inverse trend, corroborated by van der Spoel^33^ which shows hydrogen bond lifetimes decreasing from 3.638 ps for water to 8.229 ps for 1-pentanol. The increased stability of the water system as well as the decreasing stability of the organic alcohols systems were observed further from the RDF which clarified by the size effects of organic solvents. Solvent–solvent interaction instead show a progressively increasing trend from water (4.332 ps) to 1-pentanol (36.045 ps) systems, except for 2-propanol and 2-butanol mixtures. The low value of 2-propanol and 2-butanol as compared to water instead fits into the observation of Muller-Plathe,^65^ who found that solute–solute lifetimes are expected to be longer than their corresponding solvent–solvent interaction and this was explained by Vrhovsek *et al.*,^68^ who suggested that solvent–solvent hydrogen bonding strength for water would be weaker than organic alcohols due to longer bond length and wider O–HO–O bond angle.^68^

The analysis of the nature of the interaction energies between PEGML molecules with alcohols is further strengthened in Table 4. The intermolecular energy is split into homo (PEGML–PEGML) and hetero (PEGML–alcohol) contributions, both for Coulomb and Lennard-Jones terms. Intermolecular energies of PEGML–alcohols are surprisingly dominated by the Lennard-Jones which is greater than 90%, and the electrostatic contribution is relatively small, except for PEGML + methanol mixture which is slightly weaken by only 83% contribution. Intermolecular energies of pure PEGML (homo) is also dominated by the Lennard-Jones term in the range of (−268.5 to −380.2) kJ mol^−1^ for alcohol series. Since it is generally accepted that H-bonds are predominantly electrostatic interactions in origin, hence, we can conclude that the most significant interaction between oligomer in alcohols is clearly due to the van der Waals interaction. From the van der Waals energy values we can see that the magnitudes of interaction energies follow the order of hydrophobic chain in the alcohol series, being 1-pentanol > 2-butanol > *tert*-butanol ≈ 2-propanol > ethanol > methanol. In fact, several authors have paid their attention to clarifying the nature of intermolecular interaction between ethylene oxide oligomer and alcohol. For example, the MD simulations work done by Aparicio *et al.*^72^ who investigated the binary mixture of 1,2-dimethoxyethane (DME), the shortest and simplest ether molecule which has the comformational properties of PEG, in ethanol solution. The results reveal that the energy interaction of DME–ethanol mixture is strongly dominated by the Lennard-Jones type (88.9%), especially for the mixtures with low concentrations of DME.

### Solvent distributions

Although the interaction energy contributed from H-bond formation is very low in the binary mixtures of PEGML with alcohols and found to be insignificant by comparing with the van der Waals contribution, it is still interesting to analyze the distribution of the solvents around the oligomer and the interactions relevant to H-bonds formation through radial distribution functions (RDFs). RDFs define as the probability of finding a certain type of atom in a distance away from the center of mass. The radial distribution function or pair correlation function *g*_AB_(*r*) between particles A and B can be calculated by the following way:2
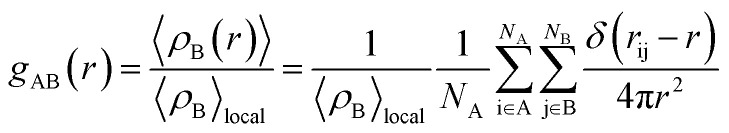
where 〈*ρ*_B_(*r*)〉 is the B particle density at distance *r* around particle A, and 〈*ρ*_B_〉_local_ is the B particle density averaged over all spheres around particles A with radius *r*_max_. The values of *r*_max_ usually is half of the box length. By convention, an intramolecular bond length of less than 0.35 nm constitutes a hydrogen bond hence it can be deduced that any RDF peaks at this range correspond to the relative frequency of hydrogen bonding (O–H interaction). Moreover, the relative width of the RDF peak describe the distance between the oxygen atom of the solvent/PEGML and the hydrogen donor in an alcohol group participating in the hydrogen bond, while the gap to the adjacent peak (if 2^nd^ peak is less than 0.35 nm) corresponds to the distance to the hydrogen/oxygen in alcohol groups not contributing directly in the hydrogen bond. Another information that can be extracted from the RDF is the coordinate number, which can be calculated from the area under the peak as well as the relative spatial volume of PEGML–solvent mixtures. A note to point is that the height of the RDF peak does not necessarily infer a higher degree of hydrogen bonding, however it does provide an indication of the presence of intermolecular hydrogen bonds between solvent terminal atoms.

**Fig. 2:**
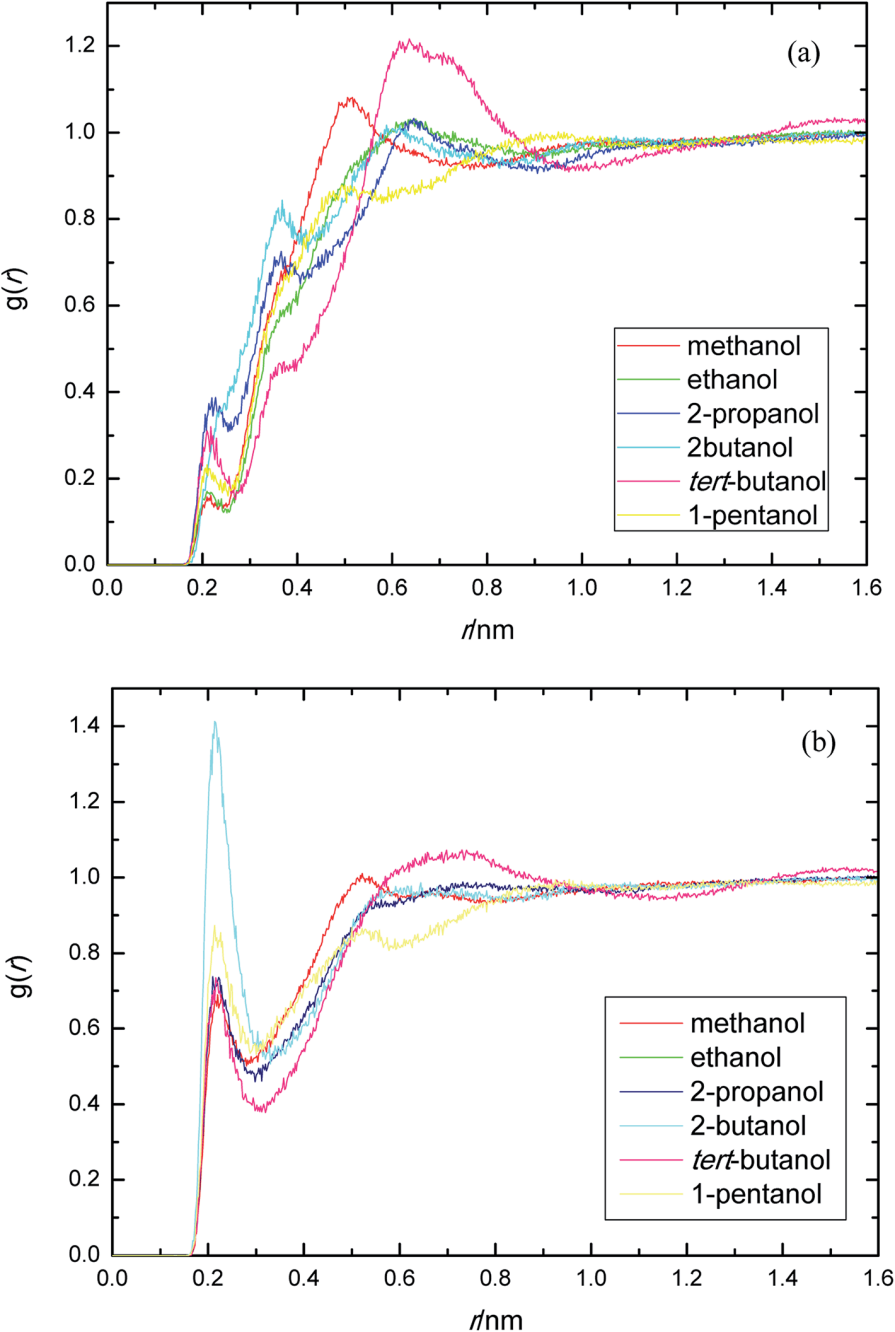
Radial distribution functions for the interactions of the proton-acceptors or donors of PEGML molecules with solvent sites: (a) OH_PEGML_–HO_solvent_; (b) HO_PEGML_–OH_solvent_.

PEGML molecule has both hydrogen bond donor and acceptor sites, which provide a number of possibilities for formation of hydrogen-bonds with the solvents. RDFs for the acceptor and donor sites for the of PEGML with alcohols are displayed in Fig. 2(a) for (OH_PEGML_–HO_solvent_) and Fig. 2(b) for (HO_PEGML_–OH_solvent_). As can be seen in Fig. 2(a), a maximal 1^st^ peak occurred at 0.21, 0.24, 0.26, 0.43, 0.28, and 0.25 nm, for methanol, ethanol, 2-propanol, 2-butanol, *tert*-butanol, and 1-pentanol, respectively. A shoulder observed for all solvents with peaks forming at 0.51 nm (methanol), 0.63 nm (ethanol), 0.64 nm (propanol), 0.62 nm (2-butanol), 0.64 nm (*tert*-butanol), and 0.50 nm (1-pentanol). It appears that, the neighbor distribution of alcohols hydroxyl atom around PEGML terminal oxygen atom does not change significantly with the variation of the alkyl chain on alcohols. As per Mendez-Morales,^73,74^ it was deduced that this shoulder phenomenon is an indicator of short-lived diatomic molecular bonding occurring. Therefore, it can be safely assumed that PEGML oxygen acceptor–solvent hydrogen donor bond does not contribute to intermolecular hydrogen bonding. Based on the comparison with Fig. 2(b) for the different RDFs of the acceptor sites, it shows that the dominant H-bonds in the binary mixtures are of the HO_PEGML_–OH_solvent_; in which the characteristic of the first peak is sharp, narrow, and more intense than that of obtained from Fig. 2(a), followed by a broad peak for all the systems studied. The first peak registered at 0.21 nm is observed in all the alcohol series which is attributed to the H-bonds between the PEGML terminal oxygen atom and the hydrogen atom of alcohols according to the H-bond criteria. The RDFs results specify that multi-solvation shells around PEGML are formed.

The RDFs displayed in Fig. 2(b) shows that the alcohol molecules are more favorable to form hydrogen bonds with the hydroxyl hydrogen atom (terminal H) of the oligomer. Even though the first sharp maxima observed at 0.21 nm shows a marginally increased H-bonds possibility, no trends were observed in the case of longer alkyl chain of alcohols. As a result, the distance at which the probability of finding PEGML terminal oxygen atom is not affected by the increasing of alkyl chain length of solvents. The highest intensity peak of the (HO_PEGML_–OH_solvent_) in Fig. 2(b) belongs to 2-butanol viewed at intensity of 1.4, while the highest one of the (OH_PEGML_–HO_solvent_) in Fig. 2(a) belongs to 2-propanol exhibits at 0.42; in which the intensity of the former is more than triple. Moreover, all (OH_PEGML_–HO_solvent_) and (HO_PEGML_–OH_solvent_) RDFs show a shoulder at the end of their first peak, which arise from the nonbonding interactions between the PEGML oxygen or hydrogen atoms and the other hydrogen or oxygen atom of alcohols that is not belongs to hydrogen bond formation. Nevertheless, it can be construed that hydroxyl hydrogen donor–solvent oxygen acceptor bond is partially responsible for the hydrogen bonding between solute and solvent.

**Fig. 3:**
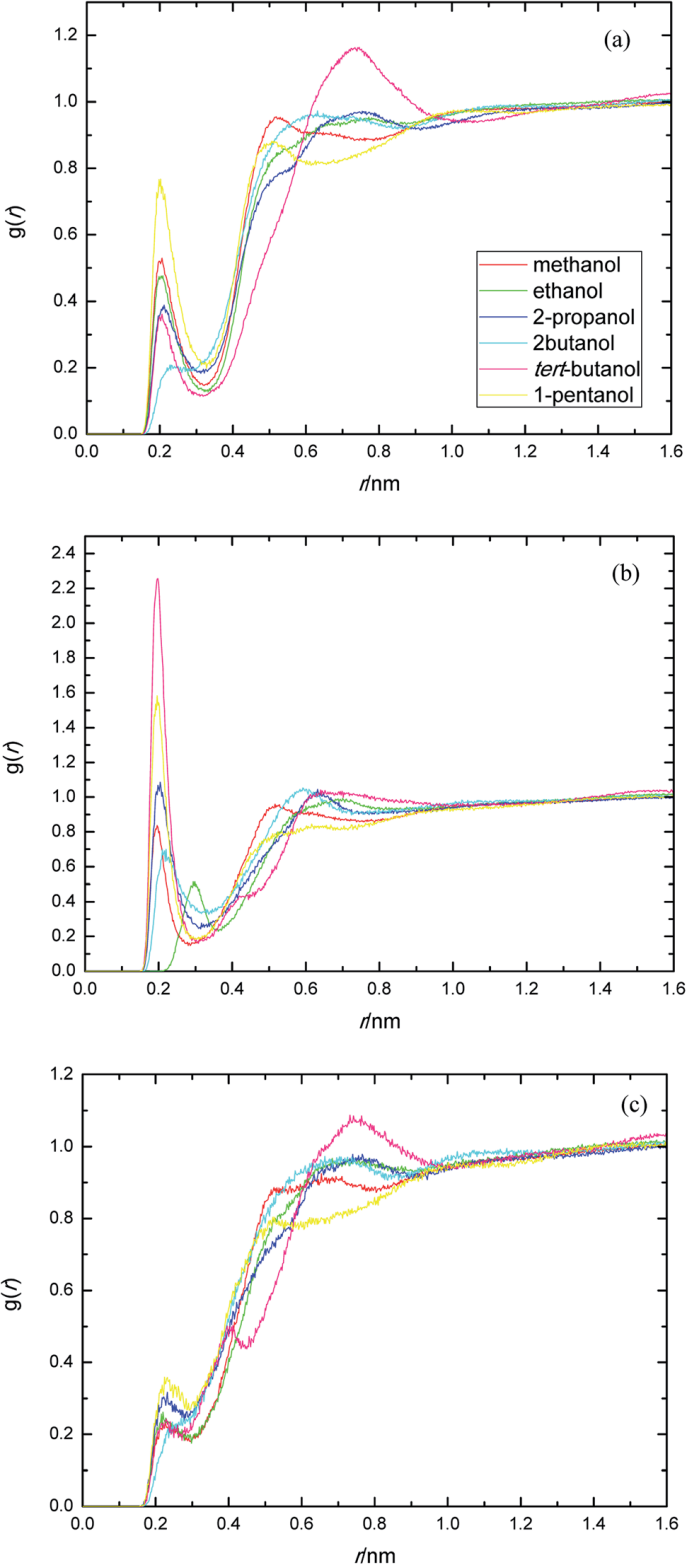
Radial distribution functions for the interactions of the proton-acceptors or donors of PEGML molecules with solvent sites: (a) O_ether_–HO_solvent_; (b) O_carbonyl_–HO_solvent_; (c) O_ester_–HO_solvent_.

**Fig. 4:**
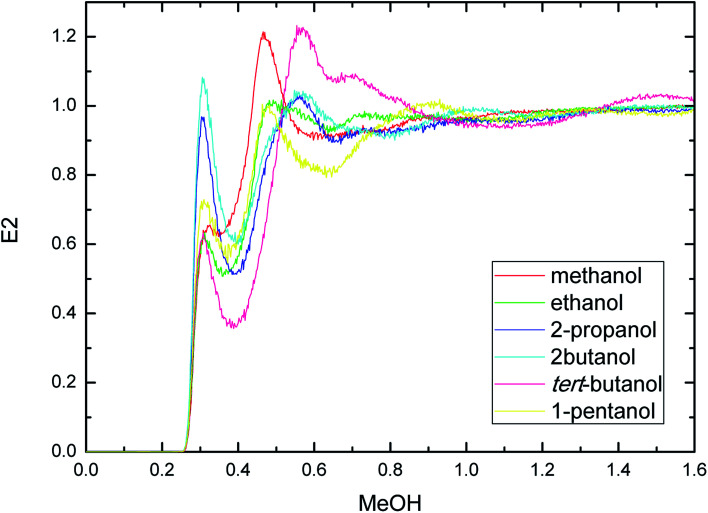
Radial distribution functions of atom O in the PEGML with respect to those in the alcohols.

The presence of ethylene glycol and ester groups in PEGML chain give more significant contribution to the H-bond formation that can not be neglected, as can be clearly seen from Fig. 3. The trend are more or less similar to those of previous RDFs figures. The H-bond formation due to the existence of ether group is displayed in Fig. 3(a) (O_ether_–HO_solvent_) while the contribution of carbonyl group is depicted in Fig. 3(b) (O_carbonyl_–HO _solvent_). In Fig. 3(a), the appearance of the first peak which categorized as H-bond formation, occurred at distance of 0.21 nm in all the alcohol series. The first minima is registered at 0.32, 0.33, 0.31, 0.30, 0.32, and 0.32 nm, respectively, for methanol, ethanol, 2-propanol, 2-butanol, *tert*-butanol, and 1-pentanol. Here, 1-pentanol appears to have the highest intensity at H-bond distance among others (0.9) while 2-butanol gives the lowest (0.2). The observance of interaction between ether group and solvent was verified by Desmukh *et al.*,^75^ whereby it was ascertained that specific hydrogen bonding network formation occurs between these two groups which increase solvability albeit by a minor factor. In addition, the formation of peaks at 0.2 nm correspond to the finding of Tritopoulou *et al.*^76^ in which they deduced that hydrogen bonding at this distance occurred due to the significantly higher mass density for glycols with hydroxyl ends *i.e.* PEGML. The H-bond formation due to the presence of double-bond oxygen attached in carbonyl group with terminal hydroxyl of solvent is shown in Fig. 3(b), where *tert*-butanol appears to have the highest possibility to form H-bond, comparing with other alcohols. Similar features are also observed from Fig. 3(c), where the O_ester_ atom in PEGML formed H-bond with HO_solvent_ at 0.21 nm. 1-Pentanol was found to have the strongest interaction among other alcohols. The results obtained from above RDFs figures indicating a favorable expositions of the polar PEGML oxygens to the solvent molecules, and thus, it shows a good miscibility (Fig. 4).

**Fig. 5:**
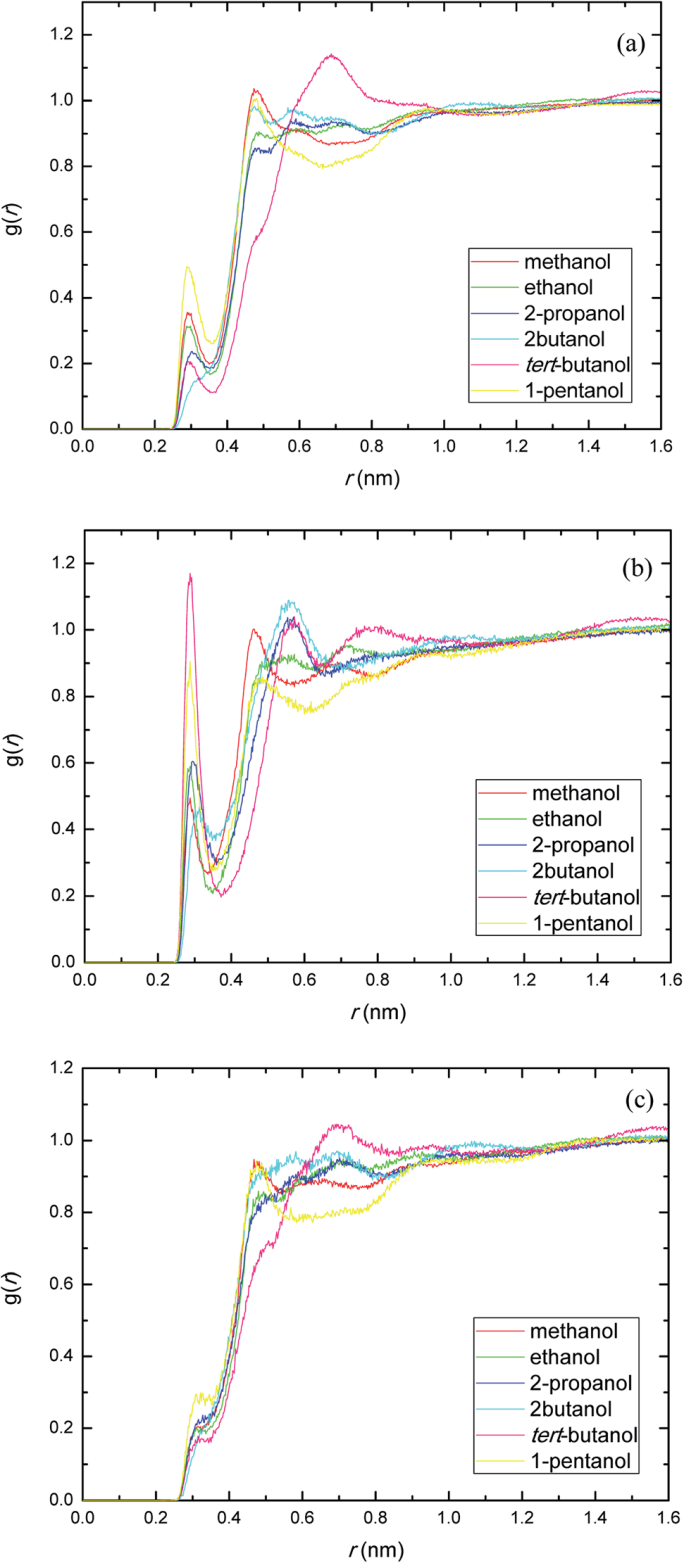
Radial distribution functions of atom O in the PEGML with respect to those in the alcohols: (a) O_ether_–OH_solvent_; (b) O_carbonyl_–OH_solvent_; (c) O_ester_–OH_solvent_.

The site–site interactions between hydroxylic oxygen, ether oxygen, carbonyl oxygen, and ester oxygen in PEGML (hydrogen bonding acceptors) with hydroxylic oxygen of alcohols are shown in Fig. 5. The RDFs of the hydroxylic oxygen atoms between PEGML and the alcohols are shown in Fig. 5, revealing a sharp first peak at 0.30 nm as seen from each binary system and its position is almost the same for this alcohol series. 2-Butanol have the highest intensity among all the systems investigated. While the site–site radial distribution function around PEGML ether oxygen atoms and alcohols showed similar tendency with the first sharp and narrow peak clearly viewed at 0.29 nm for all the alcohols. 1-Pentanol appears to have the highest intensity of the first peak, while the probability of finding atom OH in *tert*-butanol around carbonyl oxygen of PEGML is higher than other alcohols as seen in Fig. 5(b). On the contrary, the organization of site–site RDFs between ester oxygen of oligomer with hydroxylic oxygen shows a less sharp first peak as seen in Fig. 5(c), indicating a lower distribution of the alcohol molecules around the corresponding atomic group of the solute.

### Free energy of solvation

MD simulations with an aggregate length of 102.9 ns have been performed in order to study the solvation free energy of PEGML in water and in alcohols. The Gibbs free energies of solvation of a single PEGML molecule in a box of solvents at 298 K are tabulated in Table 5. As seen from this table, the trend of a gradual increase in the solvation free energy of PEGML with the increase of the dielectric constant of solvents is observed in the order of 1-pentanol > 2-butanol > *tert*-butanol > 2-propanol > ethanol > methanol > water. The presence of long chain carbon (alkyl) group in PEGML allows more favourable interactions with more hydrophobic solvents. It is worthy to mention that the solvation free energy of PEGML in water has a positive value due to the immiscibility of PEGML in aqueous solution. The negative sign of solvation free energy is attributive to two main structural features: (i) the effective packaging of both molecules (binary mixtures) which means good solubility and (ii) the H-bond formation between PEGML and alcohol molecules does not compensate the breaking of alcohol–alcohol H-bond structure in pure liquid alcohols. In addition, by comparing the solvation free energies of PEGML in alcohols with that in water, it can be concluded that PEGML prefers alcohol to water. Hence, the results explain the immiscibility of PEGML in water. More importantly, the decrease in the solvation free energy has a similar trend with that obtained from the calculated van der Waals interaction energies. The findings from the solvation free energy, therefore, justified the results indicating that the van der Waals interaction energy is the most significant contribution in the binary mixtures of PEGML with alcohols.

**Table 5:** Solvation free energy of PEGML in different solvents at 298 K^a^

Solvent	Δ*G*/kJ mol^−1^
Water	13.31 ± 0.49
Methanol	−46.28 ± 0.67
Ethanol	−55.08 ± 0.42
2-Propanol	−58.34 ± 0.59
2-Butanol	−82.36 ± 0.94
*tert*-Butanol	−80.99 ± 1.84
1-Pentanol	−84.57 ± 1.43

a The mole fraction of PEGML is 0.01 in each binary mixture.

## 4 Concluding remark

MD simulations for the mixtures containing PEGML with water or various alcohols (methanol, ethanol, 2-propanol, 2-butanol, *tert*-butanol, and 1-pentanol) have been carried out in the present work. The configuration structures consist of 10 molecules PEGML solvated in 990 molecules of solvent. The OPLS-AA force field was selected to describe PEGML and alcohols, and the SPC/E model to water. The density obtained from the simulations for each binary system agrees well with the experimental value which verifies the performance of the selected force fields. The analysis of the nature of the interaction energies between PEGML and alcohols molecules was conducted by computing average number of hydrogen bonds and strengthened by analyzing the intermolecular energies from the electrostatic (Coulomb) and van der Waals (Lennard Jones) interactions. Surprisingly, intermolecular energies for PEGML–alcohols were dominated by the Lennard-Jones, while the electrostatic contributions are only 17% or less of the Lennard-Jones for the systems investigated. Hence, the most significant interaction between the oligomer and the alcohols is clearly due to the van der Waals interaction. The distribution of the solvents around the oligomer and the interactions relevant to H-bonds formation were observed through radial distribution functions. The solvation free energy was also calculated employing the free energy perturbation approach. Trend of a gradual increase in the solvation free energy of PEGML with the increase of the dielectric constant of solvents was observed in the order of 1-pentanol > 2-butanol > *tert*-butanol > 2-propanol > ethanol > methanol > water, which has a similar tendency with those obtained from the van der Waals interaction energy. These findings, therefore, justified the results from the van der Waals interaction energy as the most significant contribution in the binary mixtures of PEGML with alcohols.

## Conflicts of interest

There are no conflics to declare.

## Supplementary Material

RA-010-C9RA09688D-s001
