# Supplementary material for: Insight into structural properties of polyethylene glycol monolaurate in water and alcohols from molecular dynamics studies
Source: RSC Adv. 2020 Jun 8;10(37):21760–71. doi: 10.1039/c9ra09688d (PMC9054503; doi:10.1039/c9ra09688d)
Supplement: RA-010-C9RA09688D-s001 [file RA-010-C9RA09688D-s001.pdf]

Electronic Supplementary Material (ESI) for RSC Advances.
This journal is © The Royal Society of Chemistry 2020

Supplementary Information

Insight into structural properties of polyethylene glycol monolaurate in water alcohols from molecular dynamics studies

Ianatul Khoiroh,[a*] Sze Ying Lee[b], Mohsen Pirdashti[c], and Ming-Jer Lee[d]

[a] Department of Chemical & Environmental Engineering, University of Nottingham Malaysia Campus, Jalan Broga, 43500 Semenyih, Selangor Darul Ehsan, Malaysia.

[b] Department of Chemical Engineering, Faculty of Engineering and Science, Universiti Tunku Abdul Rahman, Sungai Long Campus, Kajang 43000, Selangor, Malaysia.

[c] Chemical Engineering Department, Faculty of Engineering, Shomal University, Amol, PO Box 731, Iran.

[d] Department of Chemical Engineering, National Taiwan University of Science and Technology, 43 Keelung Road, Section 4, Taipei 106-07, Taiwan.

Table S.1
Include file for topology (itp file) of PEGML and the calculated atomic charges by using AM1-BCC. Atomic numbering is given in Figure S.1.

```
[ moleculetype ]
; Name      nrexcl
  PEGML       3

[ atoms ]
;  nr  type    resnr residue  atom cgnr     charge    mass
    1 opls_490  1     ML      C1     1    -0.0141079
    2 opls_182  1     ML      C2     2    -0.0213862
    3 opls_180  1     ML      O1     2    -0.2826303
    4 opls_465  1     ML      C3     3     0.3040785
    5 opls_136  1     ML      C4     4    -0.1545249
    6 opls_136  1     ML      C5     5    -0.1549852
    7 opls_136  1     ML      C6     6    -0.1592751
    8 opls_136  1     ML      C7     7    -0.1567743
    9 opls_136  1     ML      C8     8    -0.1579672
   10 opls_136  1     ML      C9     9    -0.1574738
   11 opls_136  1     ML      C10   10    -0.1576485
   12 opls_136  1     ML      C11   11    -0.1576251
   13 opls_136  1     ML      C12   12    -0.1579151
   14 opls_136  1     ML      C13   13    -0.1586728
   15 opls_135  1     ML      C14   14    -0.2105497
   16 opls_466  1     ML      O2     3    -0.3536118
   17 opls_467  1     ML      O3     3    -0.2813521
   18 opls_182  1     ML      C15   15    -0.0205762
   19 opls_182  1     ML      C16   16    -0.0207722
   20 opls_180  1     ML      O4    16    -0.2866073
   21 opls_182  1     ML      C17   17    -0.0207861
   22 opls_182  1     ML      C18   18    -0.0203461
   23 opls_180  1     ML      O5    18    -0.2861399
   24 opls_182  1     ML      C19   19    -0.0204555
   25 opls_182  1     ML      C20   20    -0.0203821
   26 opls_180  1     ML      O6    20    -0.2877822
   27 opls_182  1     ML      C21   21    -0.0207379
   28 opls_157  1     ML      C22   22    -0.0229443
   29 opls_154  1     ML      O7    22    -0.3297824
   30 opls_469  1     ML      H1     1     0.1055632
   31 opls_469  1     ML      H2     1     0.1053012
   32 opls_185  1     ML      H3     2     0.0803985
   33 opls_185  1     ML      H4     2     0.0805453
   34 opls_140  1     ML      H5     4     0.1200091
   35 opls_140  1     ML      H6     4     0.1199183
   36 opls_140  1     ML      H7     5     0.0953492
   37 opls_140  1     ML      H8     5     0.0948379
   38 opls_140  1     ML      H9     6     0.0801070
   39 opls_140  1     ML      H10    6     0.0800527
   40 opls_140  1     ML      H11    7     0.0812427
   41 opls_140  1     ML      H12    7     0.0812498
   42 opls_140  1     ML      H13    8     0.0793930
   43 opls_140  1     ML      H14    8     0.0793732
   44 opls_140  1     ML      H15    9     0.0793988
   45 opls_140  1     ML      H16    9     0.0794083
```

```
   46  opls_140  1     ML       H17   10     0.0790358
   47  opls_140  1     ML       H18   10     0.0790279
   48  opls_140  1     ML       H19   11     0.0788398
   49  opls_140  1     ML       H20   11     0.0788449
   50  opls_140  1     ML       H21   12     0.0788458
   51  opls_140  1     ML       H22   12     0.0788427
   52  opls_140  1     ML       H23   13     0.0777308
   53  opls_140  1     ML       H24   13     0.0777341
   54  opls_140  1     ML       H25   14     0.0719831
   55  opls_140  1     ML       H26   14     0.0718759
   56  opls_140  1     ML       H27   14     0.0718732
   57  opls_185  1     ML       H28   15     0.0817593
   58  opls_185  1     ML       H29   15     0.0816882
   59  opls_185  1     ML       H30   16     0.0829934
   60  opls_185  1     ML       H31   16     0.0830945
   61  opls_185  1     ML       H32   17     0.0823998
   62  opls_185  1     ML       H33   17     0.0823872
   63  opls_185  1     ML       H34   18     0.0814915
   64  opls_185  1     ML       H35   18     0.0815223
   65  opls_185  1     ML       H36   19     0.0814240
   66  opls_185  1     ML       H37   19     0.0814311
   67  opls_185  1     ML       H38   20     0.0819437
   68  opls_185  1     ML       H39   20     0.0819366
   69  opls_185  1     ML       H40   21     0.0823078
   70  opls_185  1     ML       H41   21     0.0822967
   71  opls_140  1     ML       H42   22     0.0749899
   72  opls_140  1     ML       H43   22     0.0750044
   73  opls_155  1     ML       H44   22     0.2042815

 [ bonds ]
; ai  aj funct   b0    kb
  1     2   1
  1    17   1
  1    30   1
  1    31   1
  2     3   1
  2    32   1
  2    33   1
  3    18   1
  4     5   1
  4    16   1
  4    17   1
  5     6   1
  5    34   1
  5    35   1
  6     7   1
  6    36   1
  6    37   1
  7     8   1
  7    38   1
  7    39   1
  8     9   1
  8    40   1
  8    41   1
  9    10   1
  9    42   1
```

```
   9    43   1
   10   11   1
   10   44   1
   10   45   1
   11   12   1
   11   46   1
   11   47   1
   12   13   1
   12   48   1
   12   49   1
   13   14   1
   13   50   1
   13   51   1
   14   15   1
   14   52   1
   14   53   1
   15   54   1
   15   55   1
   15   56   1
   18   19   1
   18   57   1
   18   58   1
   19   20   1
   19   59   1
   19   60   1
   20   21   1
   21   22   1
   21   61   1
   21   62   1
   22   23   1
   22   63   1
   22   64   1
   23   24   1
   24   25   1
   24   65   1
   24   66   1
   25   26   1
   25   67   1
   25   68   1
   26   27   1
   27   28   1
   27   69   1
   27   70   1
   28   29   1
   28   71   1
   28   72   1
   29   73   1

[ angles ]
;  ai   aj   ak    funct    th0    cth
    1    2    3    1
    1    2   32    1
    1    2   33    1
    1   17    4    1
    2    1   17    1
    2    1   30    1
```

2	1	31	1
2	3	18	1
3	2	32	1
3	2	33	1
3	18	19	1
3	18	57	1
3	18	58	1
5	4	16	1
5	4	17	1
5	6	7	1
5	6	36	1
5	6	37	1
6	5	34	1
6	5	35	1
6	7	8	1
6	7	38	1
6	7	39	1
7	6	36	1
7	6	37	1
7	8	9	1
7	8	40	1
7	8	41	1
8	7	38	1
8	7	39	1
8	9	10	1
8	9	42	1
8	9	43	1
9	8	40	1
9	8	41	1
9	10	11	1
9	10	44	1
9	10	45	1
10	9	42	1
10	9	43	1
10	11	12	1
10	11	46	1
10	11	47	1
11	10	44	1
11	10	45	1
11	12	13	1
11	12	48	1
11	12	49	1
12	11	46	1
12	11	47	1
12	13	14	1
12	13	50	1
12	13	51	1
13	12	48	1
13	12	49	1
13	14	15	1
13	14	52	1
13	14	53	1
14	13	50	1
14	13	51	1
14	15	54	1
14	15	55	1

14	15	56	1
15	14	52	1
15	14	53	1
16	4	17	1
17	1	30	1
17	1	31	1
18	19	20	1
18	19	59	1
18	19	60	1
19	18	57	1
19	18	58	1
19	20	21	1
20	19	59	1
20	19	60	1
20	21	22	1
20	21	61	1
20	21	62	1
21	22	23	1
21	22	63	1
21	22	64	1
22	21	61	1
22	21	62	1
22	23	24	1
23	22	63	1
23	22	64	1
23	24	25	1
23	24	65	1
23	24	66	1
24	25	26	1
24	25	67	1
24	25	68	1
25	24	65	1
25	24	66	1
25	26	27	1
26	25	67	1
26	25	68	1
26	27	28	1
26	27	69	1
26	27	70	1
27	28	29	1
27	28	71	1
27	28	72	1
28	27	69	1
28	27	70	1
28	29	73	1
29	28	71	1
29	28	72	1
30	1	31	1
32	2	33	1
34	5	35	1
36	6	37	1
38	7	39	1
40	8	41	1
42	9	43	1
44	10	45	1
46	11	47	1

```
   48   12   49   1
   50   13   51   1
   52   14   53   1
   54   15   55   1
   54   15   56   1
   55   15   56   1
   57   18   58   1
   59   19   60   1
   61   21   62   1
   63   22   64   1
   65   24   66   1
   67   25   68   1
   69   27   70   1
   71   28   72   1

[ dihedrals ]
; ai   aj ak al  funct   ph0  cp  mult
   17    1    2    3    3
   30    1    2    3    3
   31    1    2    3    3
   17    1    2   32    3
   30    1    2   32    3
   31    1    2   32    3
   17    1    2   33    3
   30    1    2   33    3
   31    1    2   33    3
    2    1   17    4    3
   30    1   17    4    3
   31    1   17    4    3
    1    2    3   18    3
   32    2    3   18    3
   33    2    3   18    3
    2    3   18   19    3
    2    3   18   57    3
    2    3   18   58    3
    6    5    4   17    3
   34    5    4   17    3
   35    5    4   17    3
    4    5    6    7    3
   34    5    6    7    3
   35    5    6    7    3
    4    5    6   36    3
   34    5    6   36    3
   35    5    6   36    3
    4    5    6   37    3
   34    5    6   37    3
   35    5    6   37    3
    5    6    7    8    3
   36    6    7    8    3
   37    6    7    8    3
    5    6    7   38    3
   36    6    7   38    3
   37    6    7   38    3
    5    6    7   39    3
   36    6    7   39    3
   37    6    7   39    3
```

6	7	8	9	3
38	7	8	9	3
6	7	8	40	3
38	7	8	40	3
39	7	8	40	3
6	7	8	41	3
38	7	8	41	3
39	7	8	41	3
7	8	9	10	3
40	8	9	10	3
41	8	9	10	3
7	8	9	42	3
40	8	9	42	3
41	8	9	42	3
7	8	9	43	3
40	8	9	43	3
8	9	10	11	3
42	9	10	11	3
43	9	10	11	3
8	9	10	44	3
42	9	10	44	3
43	9	10	44	3
8	9	10	45	3
42	9	10	45	3
43	9	10	45	3
9	10	11	12	3
44	10	11	12	3
45	10	11	12	3
9	10	11	46	3
44	10	11	46	3
45	10	11	46	3
9	10	11	47	3
44	10	11	47	3
45	10	11	47	3
10	11	12	13	3
46	11	12	13	3
47	11	12	13	3
10	11	12	48	3
46	11	12	48	3
47	11	12	48	3
10	11	12	49	3
46	11	12	49	3
47	11	12	49	3
48	12	13	14	3
49	12	13	14	3
11	12	13	50	3
48	12	13	50	3
49	12	13	50	3
11	12	13	51	3
48	12	13	51	3
49	12	13	51	3
12	13	14	15	3
50	13	14	15	3
51	13	14	15	3
12	13	14	52	3
50	13	14	52	3

51	13	14	52	3
12	13	14	53	3
50	13	14	53	3
51	13	14	53	3
13	14	15	54	3
52	14	15	54	3
53	14	15	54	3
13	14	15	55	3
52	14	15	55	3
53	14	15	55	3
52	14	15	56	3
53	14	15	56	3
3	18	19	20	3
57	18	19	20	3
58	18	19	20	3
3	18	19	59	3
57	18	19	59	3
58	18	19	59	3
3	18	19	60	3
57	18	19	60	3
58	18	19	60	3
18	19	20	21	3
59	19	20	21	3
60	19	20	21	3
19	20	21	22	3
19	20	21	61	3
19	20	21	62	3
20	21	22	23	3
61	21	22	23	3
62	21	22	23	3
20	21	22	63	3
61	21	22	63	3
62	21	22	63	3
20	21	22	64	3
61	21	22	64	3
62	21	22	64	3
21	22	23	24	3
63	22	23	24	3
64	22	23	24	3
22	23	24	25	3
22	23	24	65	3
22	23	24	66	3
23	24	25	26	3
65	24	25	26	3
23	24	25	67	3
65	24	25	67	3
66	24	25	67	3
23	24	25	68	3
65	24	25	68	3
66	24	25	68	3
67	25	26	27	3
68	25	26	27	3
25	26	27	28	3
25	26	27	69	3
25	26	27	70	3
26	27	28	29	3

```
   69   27   28   29    3
   70   27   28   29    3
   26   27   28   71    3
   69   27   28   71    3
   70   27   28   71    3
   26   27   28   72    3
   70   27   28   72    3
   27   28   29   73    3
   71   28   29   73    3
   72   28   29   73    3

[ dihedrals ]
#define improper_O_C_X_Y

[ pairs ]
;  ai   aj  funct
    3   17   1
    3   30   1
    3   31   1
   32   17   1
   32   30   1
   32   31   1
   33   17   1
   33   30   1
   33   31   1
    4    2   1
    4   30   1
    4   31   1
   18    1   1
   18   32   1
   18   33   1
   19    2   1
   57    2   1
   58    2   1
   17    6   1
   17   34   1
   17   35   1
    7    4   1
    7   34   1
    7   35   1
   36    4   1
   36   34   1
   36   35   1
   37    4   1
   37   34   1
   37   35   1
    8    5   1
    8   36   1
    8   37   1
   38    5   1
   38   36   1
   38   37   1
   39    5   1
   39   36   1
   39   37   1
    9    6   1
```

9	38	1
9	39	1
40	6	1
40	38	1
40	39	1
41	6	1
41	38	1
41	39	1
10	7	1
10	40	1
10	41	1
42	7	1
42	40	1
42	41	1
43	7	1
43	40	1
43	41	1
11	8	1
11	42	1
11	43	1
44	8	1
44	42	1
44	43	1
45	8	1
45	42	1
45	43	1
12	9	1
12	44	1
12	45	1
46	9	1
46	44	1
46	45	1
47	9	1
47	44	1
47	45	1
13	10	1
13	46	1
13	47	1
48	10	1
48	46	1
48	47	1
49	10	1
49	46	1
49	47	1
14	11	1
14	48	1
14	49	1
50	11	1
50	48	1
50	49	1
51	11	1
51	48	1
51	49	1
15	12	1
15	50	1
15	51	1

52	12	1
52	50	1
52	51	1
53	12	1
53	50	1
53	51	1
54	13	1
54	52	1
54	53	1
55	13	1
55	52	1
55	53	1
56	13	1
56	52	1
56	53	1
20	3	1
20	57	1
20	58	1
59	3	1
59	57	1
59	58	1
60	3	1
60	57	1
60	58	1
21	18	1
21	59	1
21	60	1
22	19	1
61	19	1
62	19	1
23	20	1
23	61	1
23	62	1
63	20	1
63	61	1
63	62	1
64	20	1
64	61	1
64	62	1
24	21	1
24	63	1
24	64	1
25	22	1
65	22	1
66	22	1
26	23	1
26	65	1
26	66	1
67	23	1
67	65	1
67	66	1
68	23	1
68	65	1
68	66	1
27	24	1
27	67	1

27	68	1
28	25	1
69	25	1
70	25	1
29	26	1
29	69	1
29	70	1
71	26	1
71	69	1
71	70	1
72	26	1
72	69	1
72	70	1
73	27	1
73	71	1
73	72	1

Table S.2
The calculated atomic charges of PEGML by using RESP.

```
[ moleculetype ]
; Name      nrexcl
  PEGML       3

[ atoms ]
; nr  type   resnr residue  atom  cgnr   charge     mass
    1  opls_490  1  ML  C1     1     0.3691043962    12.011
    2  opls_182  1  ML  C2     2     0.4689230369    12.011
    3  opls_180  1  ML  O1     2    -0.8364129330    15.9994
    4  opls_465  1  ML  C3     3     1.2539006151    12.011
    5  opls_136  1  ML  C4     4    -0.6718680437    12.011
    6  opls_136  1  ML  C5     5     0.3319010221    12.011
    7  opls_136  1  ML  C6     6     0.0442016085    12.011
    8  opls_136  1  ML  C7     7    -0.1018270467    12.011
    9  opls_136  1  ML  C8     8     0.1084576434    12.011
   10  opls_136  1  ML  C9     9     0.0747121790    12.011
   11  opls_136  1  ML  C10   10     0.0550785378    12.011
   12  opls_136  1  ML  C11   11     0.0342192661    12.011
   13  opls_136  1  ML  C12   12     0.0147417429    12.011
   14  opls_136  1  ML  C13   13     0.1658651316    12.011
   15  opls_135  1  ML  C14   14    -0.2370784739    12.011
   16  opls_466  1  ML  O2     3    -0.7500293071    15.9994
   17  opls_467  1  ML  O3     3    -0.7930093275    15.9994
   18  opls_182  1  ML  C15   15     0.4998390010    12.011
   19  opls_182  1  ML  C16   16     0.5710990373    12.011
   20  opls_180  1  ML  O4    16    -0.8779960864    15.9994
   21  opls_182  1  ML  C17   17     0.5050389716    12.011
   22  opls_182  1  ML  C18   18     0.5744649402    12.011
   23  opls_180  1  ML  O5    18    -0.8796376655    15.9994
   24  opls_182  1  ML  C19   19     0.4919900476    12.011
   25  opls_182  1  ML  C20   20     0.5817423126    12.011
   26  opls_180  1  ML  O6    20    -0.8407686412    15.9994
   27  opls_182  1  ML  C21   21     0.3934631471    12.011
   28  opls_157  1  ML  C22   22     0.4218024291    12.011
   29  opls_154  1  ML  O7    22    -0.8769199458    15.9994
   30  opls_469  1  ML  H1     1     0.0253328963    1.008
   31  opls_469  1  ML  H2     1     0.0253328963    1.008
   32  opls_185  1  ML  H3     2    -0.0206468332    1.008
   33  opls_185  1  ML  H4     2    -0.0206468332    1.008
   34  opls_140  1  ML  H5     4     0.1504141913    1.008
   35  opls_140  1  ML  H6     4     0.1504141913    1.008
   36  opls_140  1  ML  H7     5    -0.0556463752    1.008
   37  opls_140  1  ML  H8     5    -0.0556463752    1.008
   38  opls_140  1  ML  H9     6    -0.0007769637    1.008
   39  opls_140  1  ML  H10    6    -0.0007769637    1.008
   40  opls_140  1  ML  H11    7     0.0060006861    1.008
   41  opls_140  1  ML  H12    7     0.0060006861    1.008
   42  opls_140  1  ML  H13    8    -0.0377254766    1.008
   43  opls_140  1  ML  H14    8    -0.0377254766    1.008
   44  opls_140  1  ML  H15    9    -0.0266833354    1.008
```

45	opls_140	1	ML	H16	9	-0.0266833354	1.008
46	opls_140	1	ML	H17	10	-0.0276335919	1.008
47	opls_140	1	ML	H18	10	-0.0276335919	1.008
48	opls_140	1	ML	H19	11	-0.0271704691	1.008
49	opls_140	1	ML	H20	11	-0.0271704691	1.008
50	opls_140	1	ML	H21	12	-0.0135801452	1.008
51	opls_140	1	ML	H22	12	-0.0135801452	1.008
52	opls_140	1	ML	H23	13	-0.0319989990	1.008
53	opls_140	1	ML	H24	13	-0.0319989990	1.008
54	opls_140	1	ML	H25	14	0.0532157898	1.008
55	opls_140	1	ML	H26	14	0.0532157898	1.008
56	opls_140	1	ML	H27	14	0.0532157898	1.008
57	opls_185	1	ML	H28	15	-0.0430744089	1.008
58	opls_185	1	ML	H29	15	-0.0430744089	1.008
59	opls_185	1	ML	H30	16	-0.0560840798	1.008
60	opls_185	1	ML	H31	16	-0.0560840798	1.008
61	opls_185	1	ML	H32	17	-0.0427736303	1.008
62	opls_185	1	ML	H33	17	-0.0427736303	1.008
63	opls_185	1	ML	H34	18	-0.0551028934	1.008
64	opls_185	1	ML	H35	18	-0.0551028934	1.008
65	opls_185	1	ML	H36	19	-0.0431525860	1.008
66	opls_185	1	ML	H37	19	-0.0431525860	1.008
67	opls_185	1	ML	H38	20	-0.0544585624	1.008
68	opls_185	1	ML	H39	20	-0.0544585624	1.008
69	opls_185	1	ML	H40	21	-0.0242281794	1.008
70	opls_185	1	ML	H41	21	-0.0242281794	1.008
71	opls_140	1	ML	H42	22	0.0063324461	1.008
72	opls_140	1	ML	H43	22	0.0063324461	1.008
73	opls_155	1	ML	H44	22	0.4906676549	1.008

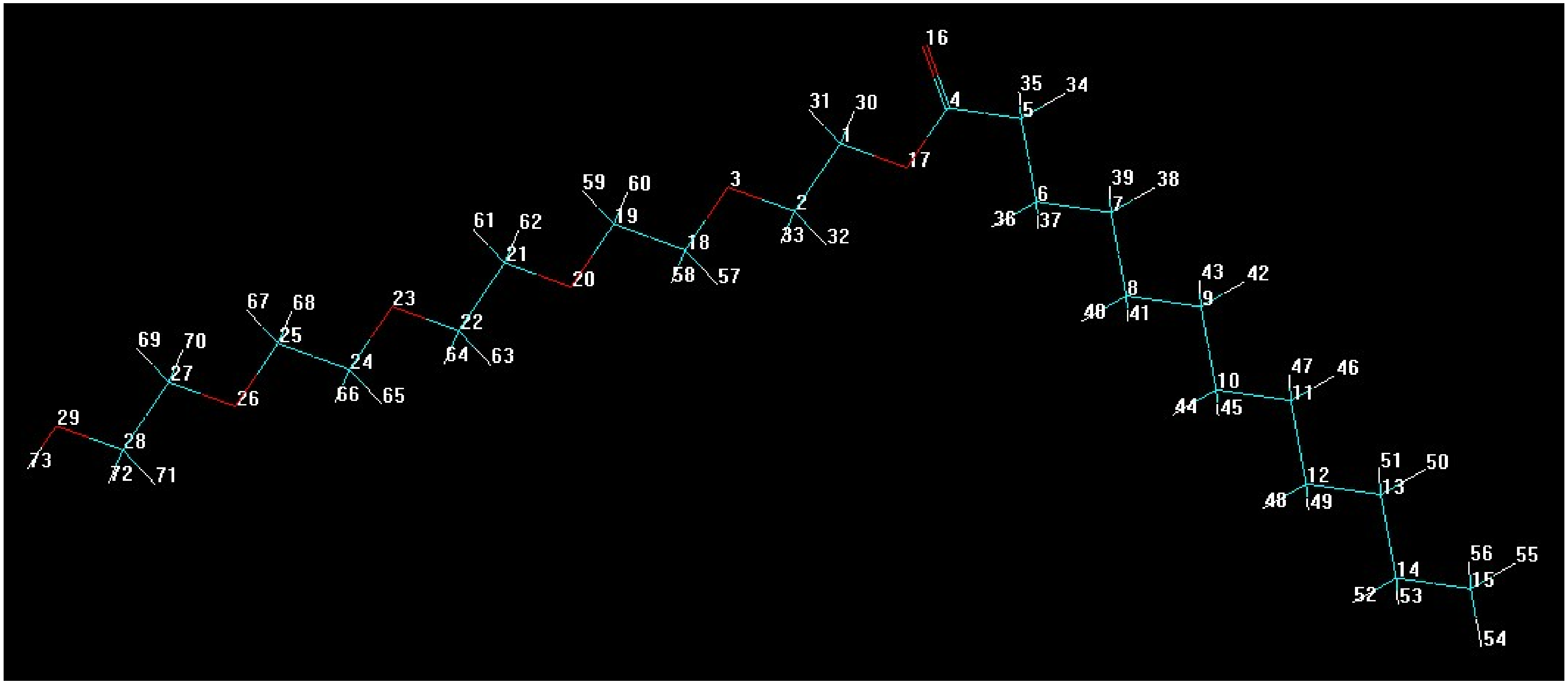

Fig. A.1 PEGML molecule with its atomic numbering. The hydrogen atoms, including the polar hydrogen, were depicted by white color, carbon atoms by blue, and oxygen atoms by red.
